# Sticking together: a chitinase-like gene involved in cell wall remodeling is required for avocado graft compatibility

**DOI:** 10.1093/plphys/kiag213

**Published:** 2026-04-15

**Authors:** Hannah Rae Thomas

**Affiliations:** Assistant Features Editor, Plant Physiology, American Society of Plant Biologists; Department of Horticulture, Zijingang Campus, Zhejiang University, Hangzhou 310058, PR China

Due to heterogeneity and slow maturation, commercial avocado (*Persea americana*) varieties, such as Haas, must be grafted to propagate (Rendón-Anaya et al. 2019). These valuable scions are grafted onto elite rootstocks that are resistant to disease and environmental stress (Ben-Ya’acov and Zilberstaine 1999). Rootstocks must also be clonally propagated through adventitious root formation, a technique to which some varieties are recalcitrant (Ernst and Holtzhausen 1978). To overcome this, a labor-intensive grafting technique known as the “Frolich Method” has been developed (Fig. 1a; Frolich and Platt 1972). This involves grafting rootstock varieties as scions onto nursing seedlings, which retain their seed. Once healed, these plants are grown in the dark to form etiolated scions. After etiolation, an unknown physiological mechanism enables etiolated rootstock cultivars to be capable of producing adventitious roots, thereby allowing them to be grafted onto fruiting varieties.

**Figure 1:**
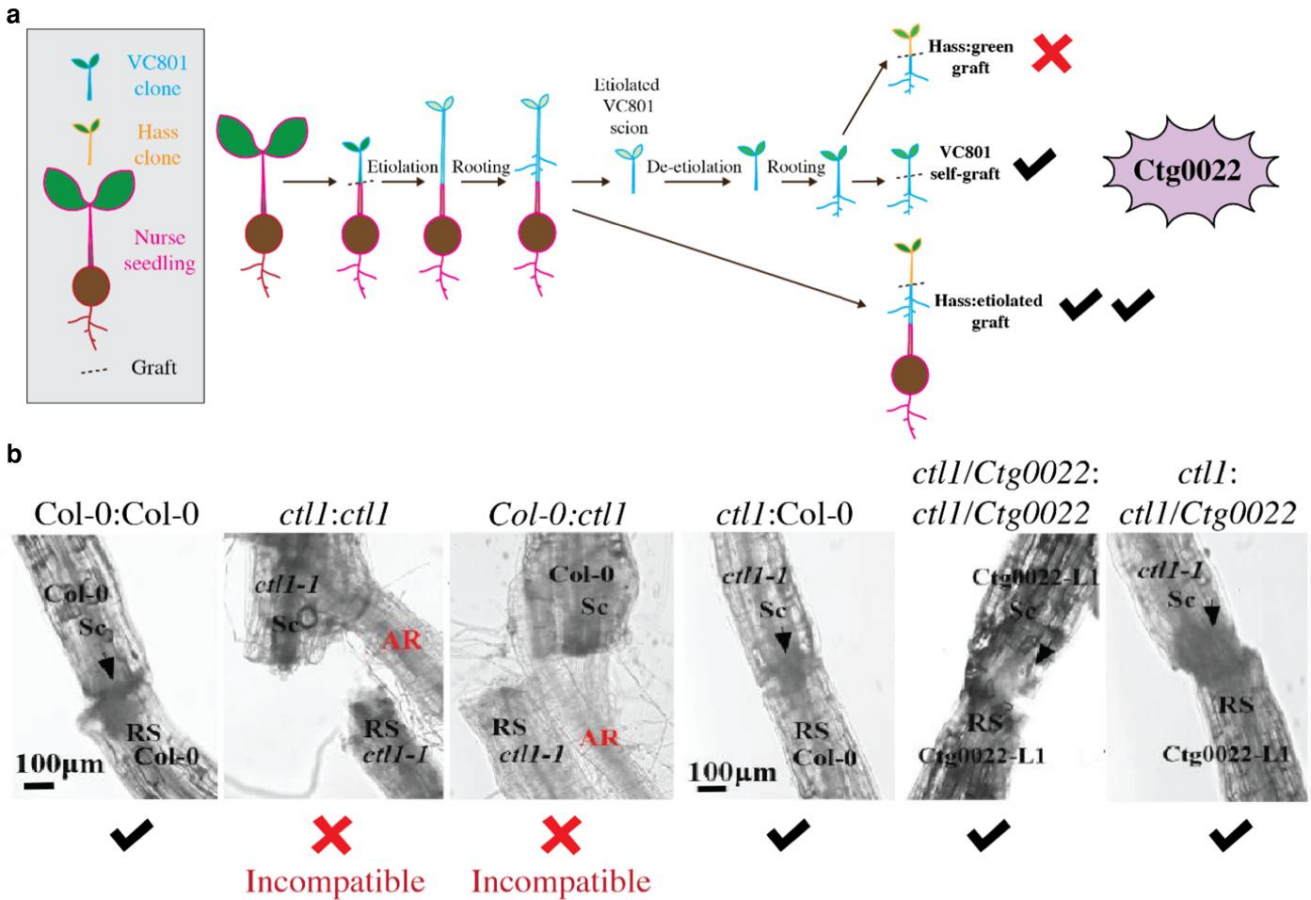
a) A flowchart depicting the use of the Frolich method used to generate the grafts studied. The rootstock VC801 is grafted onto a nurse seedling and then etiolated in darkness, thereby allowing subsequent adventitious root induction. Grafting the Hass rootstock onto this (Hass:etiolated graft) is usually successful. However, when the VC801 etiolated scion is detached and allowed to re-green, Hass:green grafts consistently fail. Interestingly, VC801 green self-grafts can heal (adapted from Duman et al. 2020). VC801 clones are depicted in blue, Hass clones are depicted in yellow, and nurse seedlings are depicted in pink. Graft sites are shown as a dashed line. Compatible grafts are annotated by a black check mark, while incompatible grafts have a red “X”. RNA-seq and RT-PCR identified *Ctg0022* as differentially expressed in the stock during compatible and incompatible grafting. b) Representative micrographs of Arabidopsis lines 7 d after grafting under a stereoscope. AtCTL1 is required in the stock for graft healing, but mutants can be complemented with the Avocado *Ctg0022* transgene (*ctl1*/*Ctg0022*). “Sc” denotes the scion, “RS” denotes the stock, “AR” denotes adventitious roots, and black arrows point to successfully healed grafts. Compatible grafts have a black check mark, while incompatible grafts have a red “X” (adapted from Dwivedi et al. 2026).

Previous work has identified the rootstock variety VC801 as disease resistant but requiring the Frolich method for use (Ben- Ya’acov and Michelson 1995). Work by Duman et al. has previously identified a method to induce adventitious roots on detached, etiolated VC801 scions by first de-etiolating the cuttings and then treating them with rooting hormones (2020). Interestingly, attempts to graft onto these green-rooted plants resulted in failed healing, which they attributed to physiological graft incompatibility.

Graft compatibility refers to the capacity of 2 plants to successfully reconnect both nonvascular and vascular tissues at the graft junction and to resume normal growth (Moore 1984). Incompatibility can manifest immediately or present weeks or months later as mechanical weakness or reduced productivity. For woody crops such as avocado, graft incompatibility poses a serious issue for growers, who risk investing time and money in failed graft combinations. There are several different types of graft incompatibility. Quince and pear are metabolically incompatible due to high levels of hydrogen cyanide in quince (Hudina et al. 2014). In contrast, tomato and pepper display a form of genetic incompatibility linked to an unknown immune response (Thomas et al. 2024). Many graft incompatibilities have been explained by taxonomic distance (Goldschmidt 2014). However, recent work has shown that certain cell wall remodeling enzymes, such as β-1,4-glucanases, may be key to unlocking inter-family graft compatibility (Notaguchi et al. 2020). Despite the necessity of grafting to propagate avocado, very little is known about the mechanism governing physiological incompatibility.

Recently in *Plant Physiology*, Sadot et al. set out to identify genetic regulators that control the ability of etiolated avocado VC801 rootstocks to graft with Hass scions (2026). By observing microscopic graft junctions and scion growth, the authors first demonstrated that etiolated VC801 rootstocks exhibited significantly greater graft success with Hass scions (Hass:etiolated) than green VC801 rootstocks (Hass:green) (Fig. 1). Next, by comparing Hass:etiolated, self-green, and Hass:green avocado grafts with a combination of RNA-seq and RT-PCR, the authors identified a differentially expressed rootstock contig containing a chitinase-like gene (*Ctg0022*). By utilizing previously published RNA-seq datasets from interspecies grafting, it was shown that chitinase-like genes are dynamically regulated in compatible grafts.

To explore the function of this specific gene, a mutant line of a predicted Arabidopsis ortholog, *CHITINASE-LIKE PROTEIN 1* (*CTL1*), was obtained and grafted. In Arabidopsis, the *ctl1* mutant showed defects in overall growth and graft healing when used as the rootstock. Despite sharing only 20.4% sequence similarity, the *Atctl1* mutant could be successfully complemented with the introduced avocado *Ctg0022* contig (Fig. 1b). Thus, AtCTL1 and Ctg0022 appear to be functionally similar.

Next, the authors explored the roles of *Ctg0022* and *AtCTL1* in cell wall dynamics during graft healing in Arabidopsis. First, they observed that *Atctl1* had reduced cellulose and increased lignin content in the root and was extremely susceptible to chemical inhibitors of microtubule and cellulose deposition. Then, using anti-pectin antibodies and RT-PCR, they observed that *Atctl1* exhibits altered pectin dynamics, with higher levels of de-esterified pectin secreted by the scion. Lastly, the authors observed delayed cell division in *Atctl1*. All these cell wall phenotypes could be either completely or partially complemented with the avocado *Ctg0022* transgene in the Arabidopsis *ctl1-1* mutant, suggesting AtCTL1 and Ctg0022 have a conserved role in cell wall remodeling, a process especially required in the rootstock during grafting.

Overall, the authors found that differences in graft success between etiolated and green-rooted VC801 rootstocks may be due to the fine-tuned regulation of *Ctg0022* and its role in cell wall patterning. Questions remain regarding how up- or downregulation of this gene affects graft healing, as comparisons with incompatible grafts showed upregulation in compatible green self-grafts and downregulation in compatible Hass:etiolated grafts. The authors hypothesize that different scion-stock combinations may regulate cell wall dynamics in distinct ways and speculate that *Ctg0022* expression, together with the physiological status of the rootstock, may synergistically regulate graft compatibility. However, the role that etiolation or distinct varieties play on the expression of this gene remains unclear. Currently, a complete understanding of *Ctg0022* in avocado is limited by the current lack of genetic resources, including mutants and transformation protocols. Recently, a similar approach to understand the role of a graft-regulating gene in a nonmodel species was employed, in which the function of *PAT1* in Norway spruce was investigated by complementing the Arabidopsis mutant with the spruce transgene (Feng et al. 2024), supporting the importance of exploring grafting in commercially grafted plant species. As more genetic tools become available in avocado, identification of graft-regulating genes is likely to become a target for rootstock breeding programs.

Recent related articles in *Plant Physiology*:

Xiong et al showed that xyloglucan endotransglucosylase/hydrolase family genes are required for the plant graft union formation through callus proliferation.

Serivichyaswat et al (2024) showed that auxin signaling in the cambium promotes tissue adhesion and vascular formation during Arabidopsis graft healing.

## Data Availability

Not applicable.
